# Efficacy and safety of natural killer cell therapy for the treatment of advanced non-small cell lung cancer: A meta-analysis and systematic review

**DOI:** 10.1007/s12026-025-09726-2

**Published:** 2025-12-06

**Authors:** Zhengnan Li, Xiu’e Wang, Shaoqing Chen, Ping Zhang, Xiujuan Wang, Xinye Ni, Chunlin Mou

**Affiliations:** 1 Research & Development Department, Sino Bio-technology Co., Ltd, Tianjin, 300384 China; 2 Department of Radiotherapy, The Affiliated Changzhou No.2 People’s Hospital of Nanjing Medical University, Changzhou Medical Center, Nanjing Medical University, Changzhou, 213003 China; 3 Department of Anatomy, School of Basic Medical Sciences, Tianjin Medical University, Tianjin, 300070 China

**Keywords:** Non-small cell lung cancer, Immunotherapy, Natural killer cells (NK), Tumor-infiltrating NK, Solid malignancies

## Abstract

To evaluate the efficacy and safety of natural killer (NK) cell therapy for the treatment of advanced non-small cell lung cancer (NSCLC). Relevant studies on NK cell therapy for advanced NSCLC were collected from PubMed, Scopus, Embase and the Cochrane Library up to August, 2024. Two reviewers independently screened the articles and retrieved the data using the Cochrane risk assessment tool. Meta-analysis was conducted with R (version 4.3.1). A total of nine trials were analyzed, including five phase 2 randomized controlled and four phase 1 studies. All were medium to high quality but exhibited high performance and attrition biases. NK cell treatment doses ranged from 1×10^9^ to 4×10^9^ cells for 2 or 3 cycles. In total, 324 patients with advanced NSCLC were included, comprising 199 who received NK cell therapy and 125 controls, all previously treated with platinum-based regimens. Meta-analysis demonstrated comparable disease control (OR = 2.68; 95% CI: 1.53–4.71) and 1-year survival (OR = 2.54; 95% CI: 1.28–5.02) between groups, with similar adverse events rates (OR = 1.37; 95% CI: 0.35–5.26). Subgroup analyses revealed no significant differences in efficacy. There was considerable heterogeneity among studies (*I*² = 0%–92.5%). Over 39 trials were registered, with only 12 marked as completed and none of the others released the outcome data. Current evidence suggests that NK cell therapy, either alone or in combination, may achieve disease control, survival outcomes and safety profiles that were comparable to existing treatments for advanced NSCLC. These findings remain exploratory and should be confirmed in larger, well-designed trials.

**Supplementary Information:**

The online version contains supplementary material available at 10.1007/s12026-025-09726-2.

## Introduction

 Natural killer (NK) cells, that originate from the bone marrow, constitute 5%–10% of peripheral blood cells [1]. These cells play a critical role in regulating both adaptive and innate immunity through an array of activating and inhibitory receptors, without recognizing neoantigens or self-antigens overexpressed on target cell surfaces [2]. In the presence of NK cells, cancer stem cells and cancer cells, expressing low major histocompatibility complex class I, cluster of differentiation (CD54) and programmed death-ligand 1, undergo necrotic and apoptotic cell death [3]. Additionally, NK cells can also engage in direct and antibody-dependent cellular cytotoxicity to combat tumors by secreting cytokines and chemokines [4].

However, in malignancy, NK cells frequently exhibit an exhausted status characterized by compromised effector functions and an altered phenotype [5]. The mechanisms underlying NK cell exhaustion in malignancies remain incompletely understood. Recent studies suggest the involvement of various negative regulatory pathways including dysregulated signaling of NK cell receptors and suppressive effects exerted by soluble factors or regulatory cells within the tumor microenvironment (TME) [6–8]. Many studies have consistently confirmed the broad antitumor effects of exogenous NK cells and demonstrated their efficacy against various cancer types [9]. Further tentative support for the efficacy of NK cells against lung cancer (LC) was derived from a laboratory investigation involving Kirsten rat sarcoma viral oncogene homolog (KRAS)-driven spontaneous LC and the implantation of cancer cells into mice [10]. Mice lacking an NK cell population exhibited a significantly higher burden of lung tumor compared to mice with NK cells. However, the limited antitumor effects of NK cells in the advanced disease stage indicates that their efficacy was mainly observed in the early stage of KRAS-driven LC in mice [11]. Recent investigations have identified NK cells infiltration in LC, associating the presence of tumor-infiltrating NK cells with a positive impact on patient prognosis [12]. Consequently, immunotherapy targeting NK cells is emerging as an appealing anticancer paradigm against LC [13].

Over the past decade, there have been significant advances in immune checkpoint and molecular targeted inhibitor therapies for the treatment of LC. However, a substantial proportion of patients are unresponsive to these treatments, underscoring the critical need for novel therapeutic approaches [5]. Fundamental research highlights the crucial role of NK cell-based immunotherapy in managing LC [14]. Nevertheless, challenges hinder the complete realization of NK cell therapy’s anti-tumor potential. First, the limited capability of NK cells to infiltrate into solid tumor tissues constrains their use in therapy [15]. As previously noted, NK cells, when present in tumors, are predominantly located in the matrix rather than directly entering the tumor tissue. Second, alterations in NK cell-activated receptors and ligands within tumors may reduce efficacy against tumors [16]. Moreover, the TME poses a significant obstacle to the unhindered movement of adoptively transferred NK cells. Despite these challenges, the increasing accumulation of data on the LC TME, immune regulatory cell populations and cancer-related changes in NK cell biology functions, as well as trafficking, are expected to enhance the effectiveness of NK cell immunotherapy [17].

Presently, numerous ongoing clinical trials are exploring targeted NK cell therapy for LC, such as immune checkpoint inhibitors plus NK cell adoptive transfer (NCT03958097), chemotherapy with NK cell adoptive transfer therapy or chimeric antigen receptor-NK cell treatment and surgery [18, 19]. Therefore, we have designed a systematic review of diverse clinical trials and applied meta-analysis to conduct an initial investigation into the safety and efficacy of NK cell-based therapies for the treatment of non-small cell lung cancer (NSCLC).

We present this article in accordance with the Preferred Reporting Items for Systematic Reviews and Meta-Analyses (PRISMA) reporting checklist.

## Methods

### Study design

A systematic review and meta-analysis were performed in accordance with the Cochrane Handbook for Systematic Reviews of Interventions. Selection and screening processes were documented using a flow diagram based on PRISMA guidelines. A data extraction form was meticulously designed to record effect size estimates from the included studies. The entire review process was carried out by one reviewer, with quality control provided by a second reviewer. Risk of bias was assessed by one reviewer, and quality control by another, using the Cochrane Risk of Bias 2 (RoB 2) tool, and any disagreements were resolved through consensus. The efficacy meta-analysis focused exclusively on studies that evaluated the benefits of NK cell therapy, either as a standalone treatment or combined with salvage options. The safety analysis included all studies that assessed the risks associated with NK cell therapy in the target population.

### Eligibility criteria

Relevant articles were retrieved from the literature based on the following eligibility criteria: (1) cohort studies (retrospective or prospective) or controlled clinical trials; (2) studies providing data on the overall survival (OS), overall response rate, progression-free survival, and the profiles of adverse events (AEs); (3) exclusion criteria which were: (1) articles not in the English language; (2) articles comprised only of abstracts; (3) preclinical studies, including animal experiments or in vitro investigations; (4) studies not involving NK cell therapy; (5) studies not involving advanced NSCLC patients; and (6) studies without sufficient data on efficacy or safety outcomes.

### Search strategy

PubMed, Embase, Web of Science and the Cochrane Library were systematically searched from database inception to August 2024. The search strategy combined Medical Subject Headings and free-text terms related to *lung cancer*, *non-small cell lung cancer (NSCLC)*, *small cell lung cancer (SCLC)*, *immunotherapy* and *natural killer (NK) cells*. Reference lists of relevant articles were also screened to identify additional eligible studies. The detailed search strings for each database are provided in the Supplementary Material.

### Study selection and quality control

The present research first involved scanning through the abstracts/titles of all potential articles retrieved during the initial literature search, and then a full-text review of each study that met the designated inclusion and exclusion criteria (*vide supra*). Afterwards, data were extracted from each article into a designed data spreadsheet for further analyses. The evaluation of each article’s outcomes adhered to a standardized procedure using RoB 2, which evaluates potential bias across five domains (randomization, deviations from intended interventions, missing outcome data, outcome measurement and selective reporting) [20]. Specifically, patient selection refers to the methods employed for recruiting participants, while both the index test and reference standard together relate to how studies were performed and data interpreted. Flow and timing were used to determine the interval between reference and index tests. Moreover, RoB 2 (https://www.riskofbias.info/welcome/rob-2-0-tool) was performed to grade the quality of the included studies into five domains through which biases might have been introduced into the results.

### Statistical analysis

Continuous variables are presented as means with 95% confidence intervals (CIs) and categorical variables as frequencies or percentages. The overall correlation between the disease control rate and 1-year OS across studies was first evaluated using weighted Pearson and Spearman rank correlation analyses. However, correlation analysis alone could not adequately reflect treatment effects. Therefore, a meta-proportion analysis was performed to assess comprehensively disease control, OS and AEs.

Each study contributed one weighted least squares data point, with weights determined by sample size. Data normality, outliers and influential points were examined through diagnostic tests and graphical analyses to ensure model robustness. A random-effects model (DerSimonian–Laird method) was used to account for between-study heterogeneity, which was evaluated using Cochran’s Q test and the *I²* statistic (*I²* > 50% indicating substantial heterogeneity). Odds ratios (ORs) and variances were calculated and missing OS variances were estimated from the corresponding 95% CIs. The results are presented as forest plots.

The ranking of interventions and *P*-scores was estimated using a frequentist framework to evaluate the probability of each intervention being most effective. To explore the relationship between disease progression and survival benefit, a novel analytical approach was introduced, defining the clinical benefit threshold as a hazard ratio ≤ 0.8 (representing at least a 1.25-fold improvement in OS), with alternative thresholds also examined. Sensitivity and specificity are illustrated using paired receiver operating characteristic curves and forest plots. Formal sensitivity or publication bias analyses were not performed due to the limited number of included studies. All statistical analyses were conducted using R software (version 4.3.1).

## Results

**Fig. 1:**
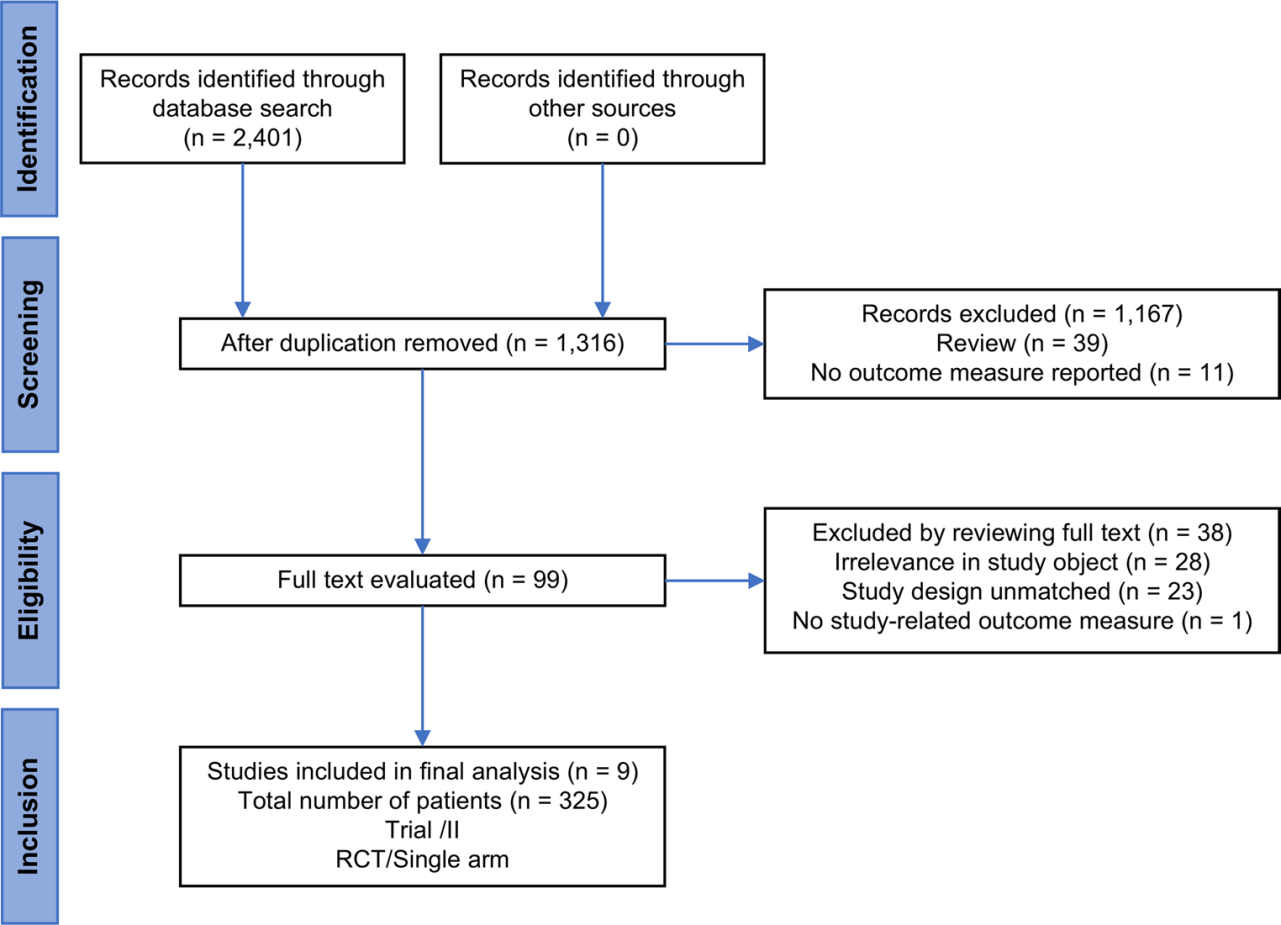
Flowchart of studies included in the meta-analysis

**Fig. 2:**
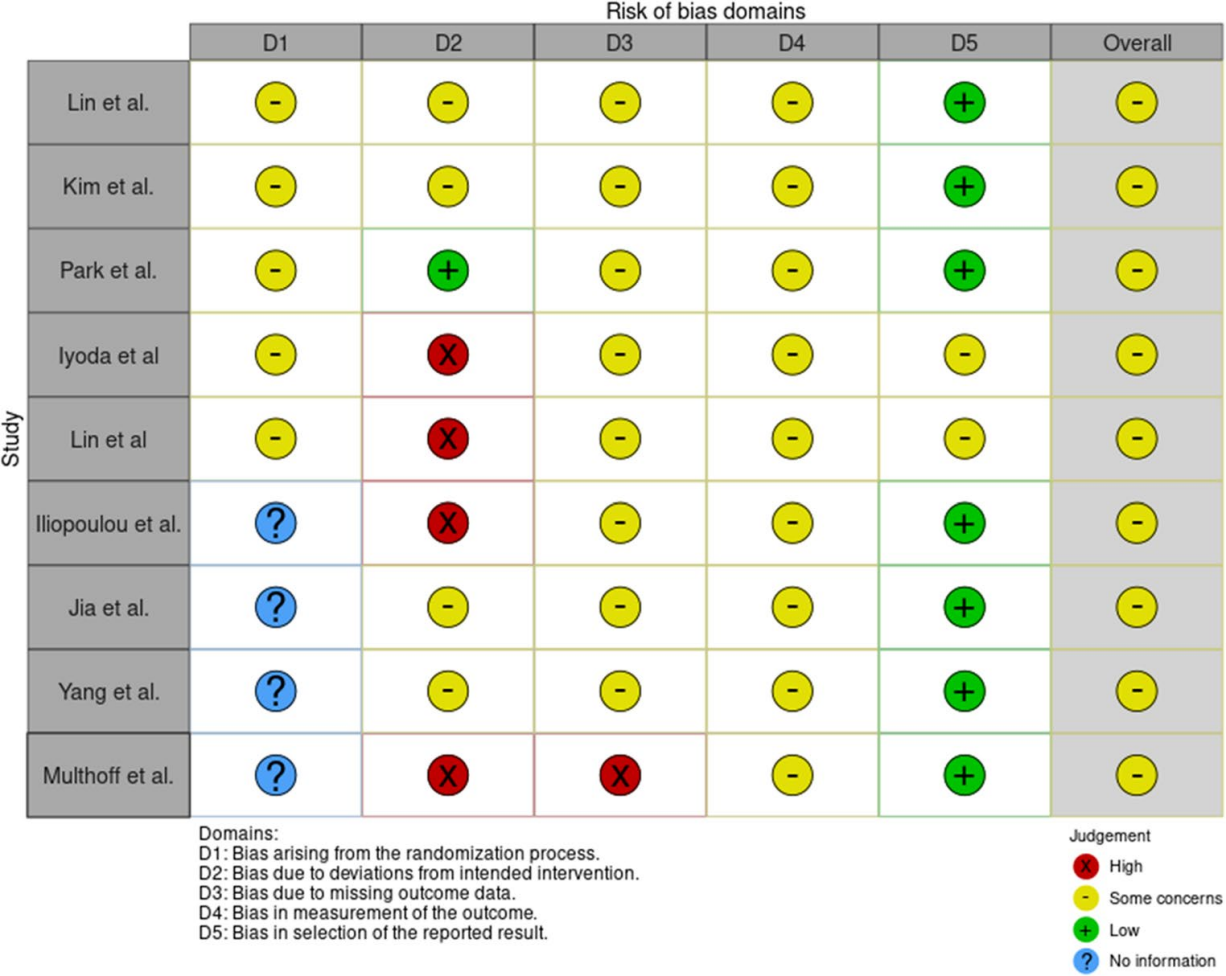
Risk of bias assessment for the included studies

The present study identified a total of 9 trials [21–29], of which 5 were phase 2 randomized controlled studies with designated control groups, while 4 were phase 1 trials (Fig. 1). All studies included in the analysis demonstrated medium to high-quality levels, but both performance and attrition biases were high in all studies (Fig. 2). More importantly, the doses of NK treatment varied from 1 × 10^9^–4 × 10^9^ for 2 or 3 cycles among different studies.

The baseline demographics of all included studies are presented in Table 1 and Table 2, which show that 324 advanced (stage IIA, IIIA, IIIB and IV) NSCLC patients were included for further analyses. Of these, 199 were assigned to either a combined or solely NK cell therapy group, while 125 cases comprised the control group. In addition, all patients from 9 studies underwent platinum-based therapy as their failed first-line treatment regimen, of whom 4 received NK cell therapy as combined regimens with programmed cell death protein 1 inhibitors, 1 with cryoablation, 1 with chemotherapy and 3 without combination therapy (Table 1). Moreover, efficacy outcomes on survival patients with disease control together with safety profiles were extracted from the included studies (Table 2).

**Table 1:** Summary of baseline demographics of included studies

Study	Number	Clinical stages	First-line therapy	Second-line therapy
Lin et al.	109	IIIB or IV	Platinum-based therapy	NK cells & pembrolizumab
Kim et al.	18	IIIB or IV	Platinum-based therapy	NK cells & pembrolizumab
Park et al.	18	IIIB or IV	Platinum-based therapy	NK cells & pembrolizumab
Iyoda et al.	56	IIA or IIB or IIIA	Platinum-based therapy	NK cells
Lin et al.	60	III or IV	Platinum-based therapy	Cryoablation & NK cells
Iliopoulou et al.	16	IIIB or IV	Platinum-based therapy	NK cells
Jia et al.	20	IIIB or IV	Platinum-based therapy	NK cells & sintilimab
Yang et al.	19	IIIB or IV	Platinum-based therapy	NK cells & docetaxel
Multhoff et al.	8	IIIB or IV	Platinum-based chemotherapy	NK cells

*NK* natural killer

**Table 2:** Summary of the efficacy and safety of NK cell therapy in the included studies

	Plus NK cell therapy				Control/historical control				Remarks
Author	Total	DCN	NS^*^	AE	Control	DCN	NS^*^	AE	
Lin et al.	55	20	43	7	54	10	30	6	Phase Ⅱ
Kim et al.	12	5	8	0	6	0	3	0	Phase Ⅱ
Park et al.	12	6	7	2	6	1	3	2	Phase Ⅱ
Iyoda et al.	27	17	26	5	29	12	26	0	Phase II
Lin et al.	30	25	30	0	30	21	30	0	Phase II
Iliopoulou et al.	16	8	9	2	0	0	0	0	Phase Ⅰ
Jia et al.	20	9	13	0	0	0	0	0	Phase Ⅰ
Yang et al.	19	5	0	3	0	0	0	0	Phase Ⅰ
Multhoff et al.	8	2	5	1	0	0	0	0	Phase Ⅰ

*AE* adverse event, *DCN* number of patients achieving disease control (complete response partial response or stable disease), *NK* natural killer, *NS* ^*^ number of surviving patients

**Fig. 3:**
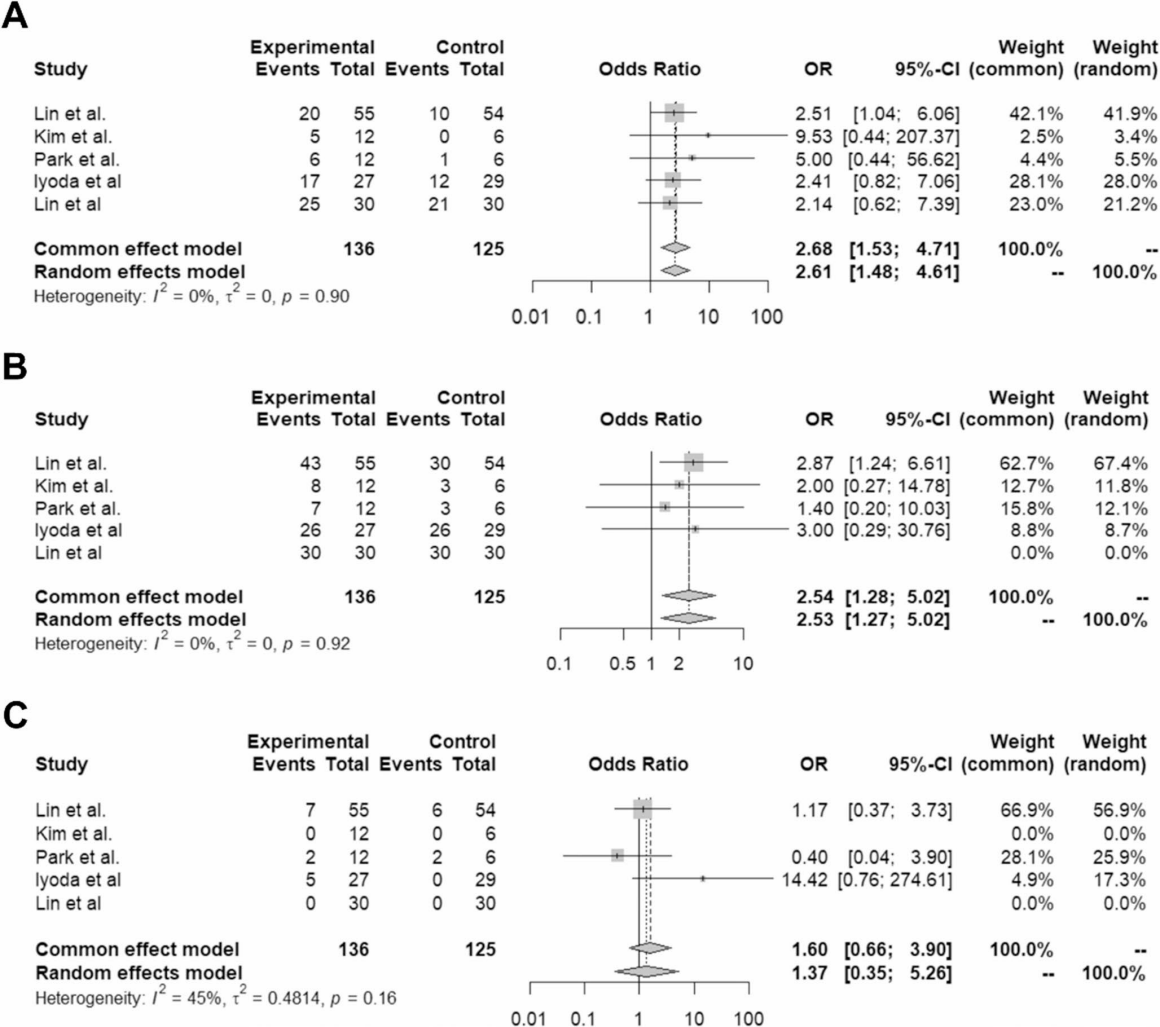
Forest plots for meta-analysis on disease control (**A**), progression-free survival (**B**), and adverse events (**C**) comparing the studies with and without NK therapy. Abbreviations: CI = confidence interval, NK = natural killer, OR = odds ratio

**Fig. 4:**
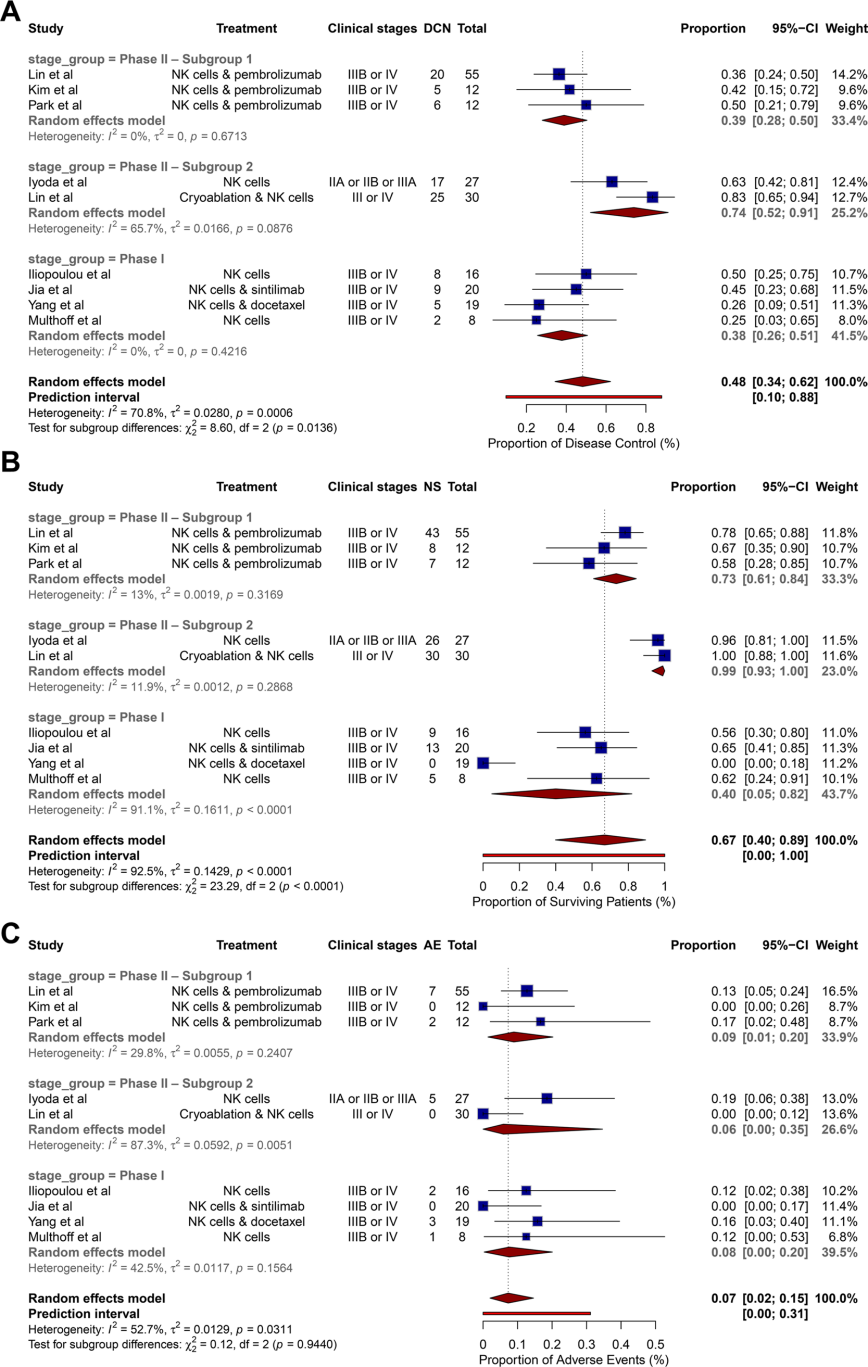
Forest plots for the subgroup analysis on disease control (**A**), progression-free survival (**B**) and adverse events (**C**), comparing the studies with NK therapy. Abbreviations: CI = confidence interval, NK = natural killer, OR = odds ratio

The meta-analysis showed that NK cell therapy achieved outcomes comparable to those of the control group and 1-year survival. Specifically, the OR for disease control was 2.68 (95% CI: 1.53–4.71) and for 1-year survival 2.54 (95% CI: 1.28–5.02) (Fig. 3A, B). AEs in the NK cell therapy group were not significantly different from those in the control group, with an OR of 1.37 (95% CI: 0.35–5.26) (Fig. 3C). Subgroup analyses based on different experimental designs showed no significant differences in efficacy across studies. For disease control, the OR was 0.48 (95% CI: 0.34–0.62); for 1-year survival, the OR was 0.67 (95% CI: 0.40–0.89) and for AEs, the OR was 0.07 (95% CI: 0.02–0.15) (Fig. 4A, B, C). However, there was considerable heterogeneity among the included studies, with *I*² values ranging from 0% to 92.5%

**Fig. 5:**
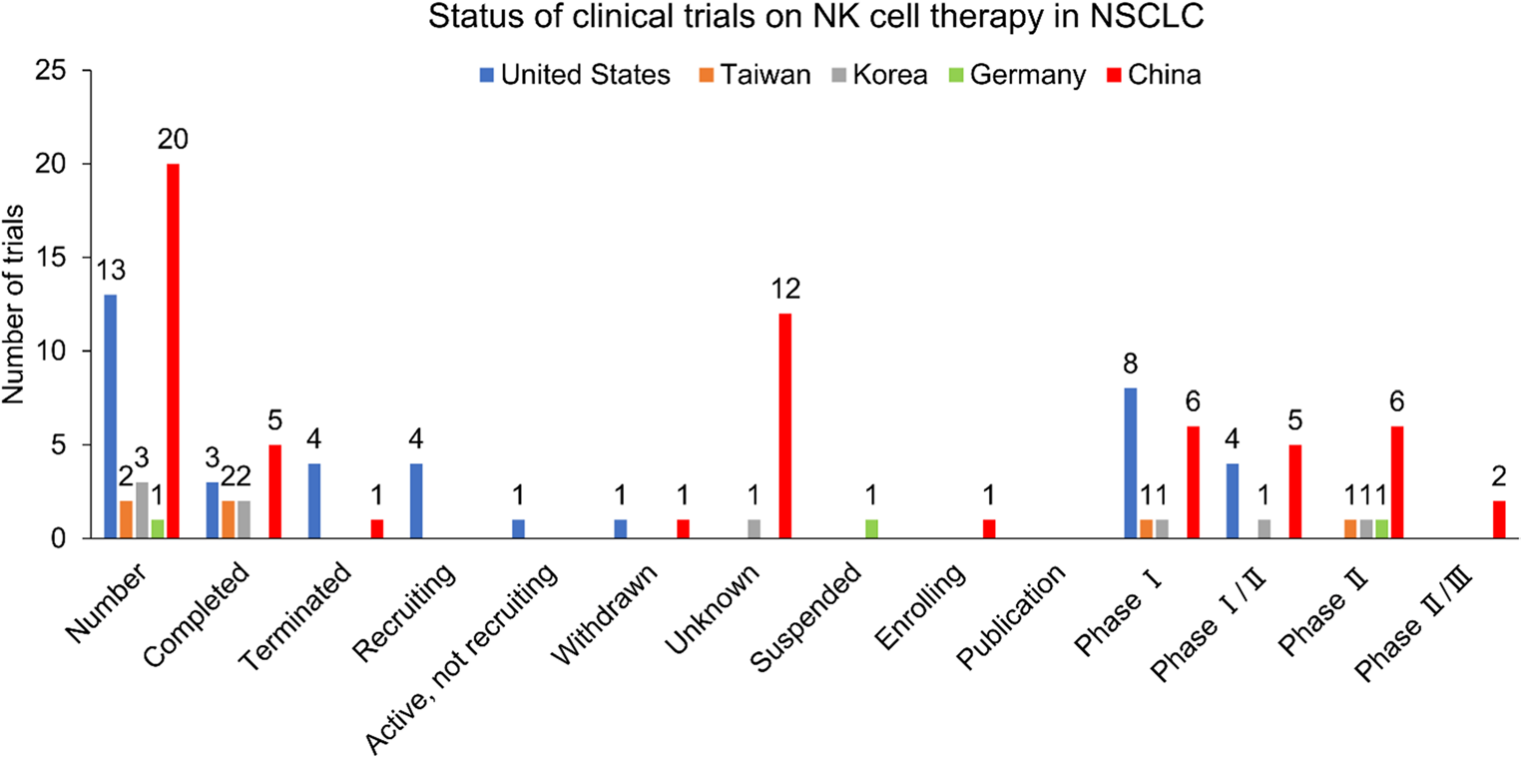
Status of the registered trials evaluating NK cell therapy for the treatment of NSCLC. Abbreviations: NK = natural killer, NSCLC = non-small cell lung cancer

Furthermore, more than 39 phase 1 or phase 2 clinical trials were registered in ClinicalTrials.gov, with only 12 updated as completed, while the status of the remaining trials was either suspended, withdrawn or unknown. None of the trials released any information or data on the investigative outcomes of NK cell therapy for the treatment of advanced NSCLC (Fig. 5).

## Discussion

Various immunotherapeutic approaches, including NK cell therapy [27], have been explored in the field of NSCLC treatments. Several phase 1 and 2 clinical trials have examined the safety and efficacy of NK cell-based therapies in patients with advanced NSCLC and the early findings suggest favorable tolerability and potential clinical activity [21–27]. In our meta-analysis, NK cell regimens demonstrated outcomes comparable to those of the controls in terms of disease control, survival and AEs. Subgroup analyses did not reveal superiority of NK cells alone or in combination, but the overall evidence supports NK therapy as a feasible strategy with an acceptable safety profile.

Adoptive transfer of high-quality activated NK cells is a common strategy in clinical trials to restore and enhance immune system functions [30, 31]. Its effectiveness depends on the methods of isolation, expansion and activation [32]. In some solid tumor studies, NK cells persisted in peripheral blood but did not consistently translate into tumor regression [33]. Potential contributing factors include receptor-ligand mismatches and the suppressive TME. These barriers may also influence outcomes in NSCLC.

Autologous NK cell administration has demonstrated safety in clinical studies [34]. Allogeneic NK cells, in contrast, often show prolonged persistence in vivo and have provided clinical benefit when combined with conventional therapies [35], while the risk of graft-versus-host disease appears low with haploidentical donors [36]. Previous research has also shown that allogeneic NK cell therapy can improve the quality of life of patients with advanced NSCLC [21]. Ex vivo expansion and cytokine stimulation (e.g., interleukin [IL]-2, IL-15) can improve activity, although persistence and reduced CD16 expression remains a limitation [37–39]. Genetic modification of NK cell lines, for example NK92-CD16, has yielded improved cytotoxicity against resistant NSCLC cells in preclinical settings [40, 41]. In addition, heat shock protein-70-activated NK cells have shown acceptable safety and preliminary signals of activity in clinical settings [27, 42, 43]. Collectively, these findings highlight both opportunities and challenges in optimizing NK cell therapy for solid tumors.

The broader experience of NK therapy for hematological malignancies further illustrates its potential. In patients with acute myeloid leukemia undergoing haploidentical stem cell transplantation, alloreactive NK cells effectively eradicated leukemic blasts and significantly improved survival [44]. These encouraging results have driven extensive investigations across various cancer types, with nearly 900 clinical trials registered globally. For solid tumors, combinations of NK cells with monoclonal antibodies, such as anti-GD2 in neuroblastoma, have produced promising outcomes [45, 46]. Continued exploration of NK-based approaches for NSCLC is therefore warranted.

Interestingly, the use of NK cells for the treatment of advanced LC ranks as the third most common treatment for solid tumors among the 63 phase 1 and 2 trials registered at ClinicalTrials.gov, with 39 specifically investigating the role of NK cells for the treatment of NSCLC. However, according to the most recent data, all of these trials are ongoing and have not reported safety or efficacy results, although 12 are reported as completed. The lack of published results may indicate potentially negative or inconclusive results for NK cell therapy for NSCLC. In addition, 27 of the 39 registered trials are currently suspended, withdrawn, terminated or have an unknown status. Disease progression is considered the primary reason for these suspensions or withdrawals, with additional factors such as loss to follow-up, poor adherence and the logistical or economic burden of treatment also contributing.

The present study had several limitations. First, the lack of randomized controlled trials limited its ability to comprehensively compare the efficacy and safety of NK cell therapy with appropriate control groups. Second, although the meta-analysis relied as much as possible on high-quality studies, such publications remain relatively scarce in this area. Third, the substantial heterogeneity observed in this meta-analysis (*I*² up to 92.5%), which may be attributed to differences in study design (randomized vs. non-randomized), disease control criteria, survival endpoints, patient characteristics (e.g., disease stage, prior treatments), intervention protocols (source and preparation of NK cells, dosing regimens) and follow-up durations. These factors likely contributed to the variability in treatment effects across the studies. Fourth, the lack of consensus on the optimal NK cell dosage and treatment protocols for NSCLC complicated the interpretation and comparison of efficacy and safety outcomes across studies. Fifth, although the present study strictly adhered to PRISMA guidelines to ensure methodological rigor and minimize potential bias, the review protocol was not registered in PROSPERO, which may to some extent affect the transparency and reproducibility of this review. Future studies with larger sample sizes and well-designed multicenter randomized controlled trials are warranted to validate further the potential of NK cell therapy as an immunotherapeutic strategy for the treatment of NSCLC.

## Conclusions

In summary, NK cell therapy, whether used alone or in combination, appears to provide comparable disease control, survival outcomes and safety profile for the treatment of patients with advanced NSCLC. However, these conclusions should be interpreted with caution, as a significant number of registered clinical trials lacked sufficient phase 1 and 2 evidence. Further well-designed studies are warranted to evaluate better the potential role of NK cell therapy for the treatment of solid malignancies such as NSCLC.

## Supplementary Information

Below is the link to the electronic supplementary material.

Supplementary Material 1 (DOCX. 16.7 KB)

## Data Availability

The data supporting this review are from previously reported studies and datasets, which have been cited.
