# Supplementary material for: Efficacy and safety of natural killer cell therapy for the treatment of advanced non-small cell lung cancer: A meta-analysis and systematic review
Source: Immunol Res. 2025 Dec 6;73(1):172. doi: 10.1007/s12026-025-09726-2 (PMC12680858; doi:10.1007/s12026-025-09726-2)
Supplement: Supplementary file 1 — Supplementary Material 1 (DOCX. 16.7 KB) [file 12026_2025_9726_MOESM1_ESM.docx]

**Search Strategies**

The search terms comprised a combination of subject words and free words, including but not limited to "lung cancer", "non-small cell lung cancer", "small cell lung cancer", "immunotherapy", "natural killer cell", "natural killer T cell", "lung cancer/immunotherapy", "non-small cell lung cancer/immunotherapy", "small cell lung cancer/immunotherapy", "lung cancer/natural killer cell", "non-small cell lung cancer/natural killer cells/immunotherapy", "small cell lung cancer/natural killer cell", "lung cancer/natural killer cells/cohort study", "lung cancer/natural killer cell/retrospective study", "lung cancer/natural killer cell/prospective study", "lung cancer/natural killer cell/clinical trial/phase I", "lung cancer/natural killer cell/clinical trial/phase II/2", "lung cancer/natural killer cell/clinical trial/phase III/3".

Using the PubMed database, as an example, the simplified search formula was ("lung cancer" OR "non-small cell lung cancer" OR "small cell lung cancer" OR "immunotherapy" OR "cell therapy" OR "natural killer cell") AND ("lung cancer, immunotherapy" OR "non-small cell lung cancer, immunotherapy" OR "small cell lung cancer, immunotherapy" OR "lung cancer, natural killer cell" OR "Non-small cell lung cancer, natural killer cells" OR "small cell lung cancer, natural killer cell") AND ("lung cancer, immunotherapy, natural killer cell" OR "non-small cell lung cancer, immunotherapy, natural killer cell" OR "small cell lung cancer, immunotherapy, natural kill cell") AND ("lung cancer, immunotherapy, natural killer cell, trial, phase I/1" OR "non-small cell lung cancer, immunotherapy, natural killer cell, trial, phase I/1" OR "small cell lung cancer, immunotherapy, natural kill cell, trial, phase I/1") AND ("lung cancer, immunotherapy, natural killer cell, trial, phase II/2" OR "non-small cell lung cancer, immunotherapy, natural killer cell, trial, phase II/2" OR “small cell lung cancer, immunotherapy, natural kill cell, trial, phase II/2”).
